# Inhibition of Syndecan-4 by therapeutic antibodies reduces TNFα dependent joint destruction in mice

**DOI:** 10.1186/ar3665

**Published:** 2012-02-09

**Authors:** Athanasios Stratis, Katja Neugebauer, Mareike Frohling, Peter Paruzel, Berno Dankbar, Corinna Wemeyer, Christoph Cromme, Lars Godmann, Jessica Bertrand, Adelheide Korb, Frank Echtermeyer, George Kollias, Thomas Pap

**Affiliations:** 1 Institute of Experimental Musculoskeletal Medicine, University Muenster, 48149, Muenster, Germany; 2 Department of Anesthesiology and Intensive Care Medicine, Medical University Hannover, 30625, Hannover, Germany; 3 Institute of Immunology, Biomedical Sciences Research Center, Vari, 16672, Greece

## Background

Syndecan-4, a member of a syndecan family of transmembrane heparansulfate proteoglycans has been recently associated with cell-matrix-adhesion, cell-migration, differentiation and proliferation, but its specific function in inflammatory pathologies remains unclear. We used the human TNFalpha transgenic mouse (hTNFtg) to analyse the expression and function of syndecan-4 in chronic-destructive-arthritis and answer the question whether inhibition of syndecan-4 by specific antibodies may prevent cartilagedestruction and/or improve the phenotype after onset of the disease in this animal model of human RA.

## Methods

Expression of syndecan-4 was investigated by immunohistochemistry in the hind-paws of 8-weeks/12-weeks old hTNFtg mice and wild type controls. In addition, synovial fibroblasts were isolated and analysed for syndecan-4-expression by RT-PCR. For functional analyses, we generated blocking-antibodies against syndecan-4. To investigate their effect on TNFalpha mediated-destructive-arthritis, hTNFtg mice were injected with the antibodies or with IgG-control twice weekly for 4-weeks in a preventive manner (age-4-to-8-weeks) and for disease treatment of joint destruction (age-8-to-12-weeks) into their hind paws. Evaluation of disease severity included clinical parameters (weight, arthritis-score, grip-strength) as well as histomorphometric analysis of toluidin-blue-stained paraffin sections.

## Results

As seen in immunohistochemistry, there was a strong expression of syndecan-4 in the synovial membranes of hTNFtg mice, whereas only negligible staining for syndecan-4 was found in synovial tissues of wild type animals. In vitro, synovial fibroblasts isolated from hTNFtg mice showed more than 30-fold higher expression of syndecan-4 than wild type controls. Administration of the anti-syndecan-4 antibodies but not of IgG-control in preventive treated 4-week-old hTNFtg mice clearly ameliorated the clinical signs of arthritis and protected the treated joints from cartilage damage. At histomorphometric analysis, this was evident for all analysed parameters but seen most prominently for area of distained cartilage.

Significantly reduced cartilage damage in the anti-syndecan-4 treated hTNFtg mice was accompanied by a striking reduction in the expression of MMP-3. The treatment with antisyndecan-4 in 8-week-old hTNFtg mice after onset of arthritis clearly ameliorated the jointdestruction, and improved cartilage-damage. The treatment also showed a clear reduction of inflammation in the paws compared to the untreated animals.

## Conclusions

Our findings indicate that syndecan-4 is involved prominently in fibroblast-mediated cartilagedamage in hTNFtg mice by regulating the exression of disease-relevant MMPs. More importantly, the data suggest that inhibition of syndecan-4 not only prevens cartilage damage, but also reduces the severity after onset of the disease.

